# Bis(propane-1,2-diammonium) benzene-1,2,4,5-tetra­carboxyl­ate dihydrate

**DOI:** 10.1107/S1600536810044065

**Published:** 2010-11-06

**Authors:** Hoda Pasdar, Maryam Majdolashrafi, Hossein Aghabozorg, Hamid Reza Khavasi

**Affiliations:** a Department of Chemistry, Islamic Azad University, North Tehran Branch, Tehran, Iran; b Department of Chemistry, Shahid Beheshti University, G. C., Evin, Tehran 1983963113, Iran

## Abstract

In the crystal of the title hydrated molecular salt, 2C_3_H_12_N_2_^2+^·C_10_H_2_O_8_^4−^·2H_2_O, the packing is stabilized by extensive N—H⋯O and O—H⋯O hydrogen-bonding inter­actions involving all three species, forming a supra­molecular three-dimensional structure. The tetraanion is generated by inversion.

## Related literature

For proton transfer systems, see: Aghabozorg *et al.* (2008 ▶); Arora & Pedireddi (2003 ▶). For related structures, see: Wang *et al.* (2005 ▶); Ma *et al.* (2005 ▶); Mrvos-Sermek *et al.* (1996 ▶); Rafizadeh *et al.* (2006 ▶).
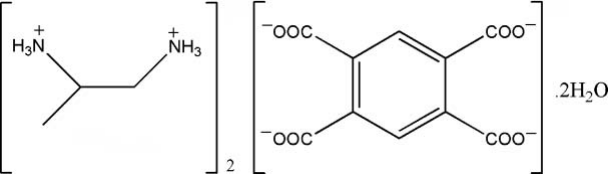


## Experimental

### 

#### Crystal data

2C_3_H_12_N_2_^2+^·C_10_H_2_O_8_^4−^·2H_2_O
*M* *_r_* = 438.44
Monoclinic, 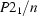

*a* = 10.427 (2) Å
*b* = 7.6955 (15) Å
*c* = 12.854 (3) Å
β = 101.61 (3)°
*V* = 1010.3 (3) Å^3^
*Z* = 2
Mo *K*α radiation
μ = 0.12 mm^−1^
*T* = 298 K
0.49 × 0.40 × 0.08 mm

#### Data collection

Bruker SMART CCD area-detector diffractometer
Absorption correction: multi-scan (*SADABS*; Sheldrick, 1998 ▶) *T*_min_ = 0.940, *T*_max_ = 0.990
8157 measured reflections
3038 independent reflections
2727 reflections with *I* > 2σ(*I*)
*R*_int_ = 0.026

#### Refinement

*R*[*F* ^2^ > 2σ(*F* ^2^)] = 0.041
*wR*(*F* ^2^) = 0.112
*S* = 1.07
3038 reflections
146 parameters
H atoms treated by a mixture of independent and constrained refinement
Δρ_max_ = 0.39 e Å^−3^
Δρ_min_ = −0.19 e Å^−3^

### 

Data collection: *SMART* (Bruker, 2007 ▶); cell refinement: *SAINT* (Bruker, 2007 ▶); data reduction: *SHELXTL* (Sheldrick, 2008 ▶); program(s) used to solve structure: *SHELXTL*; program(s) used to refine structure: *SHELXTL*; molecular graphics: *ORTEP-3 for Windows* (Farrugia, 1999 ▶); software used to prepare material for publication: *WinGX* (Farrugia, 1999 ▶).

## Supplementary Material

Crystal structure: contains datablocks I, global. DOI: 10.1107/S1600536810044065/jj2064sup1.cif

Structure factors: contains datablocks I. DOI: 10.1107/S1600536810044065/jj2064Isup2.hkl

Additional supplementary materials:  crystallographic information; 3D view; checkCIF report

## Figures and Tables

**Table 1:** Hydrogen-bond geometry (Å, °)

*D*—H⋯*A*	*D*—H	H⋯*A*	*D*⋯*A*	*D*—H⋯*A*
O*W*1—H1*W*⋯O1^i^	0.85 (2)	1.91 (2)	2.7596 (17)	172 (2)
O*W*1—H2*W*⋯O1^iv^	0.81 (2)	2.20 (2)	2.9845 (19)	167 (2)
N1—H1*A*⋯O2^ii^	0.89	1.98	2.8289 (15)	159
N1—H1*B*⋯O2^i^	0.89	1.91	2.7910 (13)	168
N1—H1*C*⋯O3^iii^	0.89	1.94	2.8130 (14)	168
N2—H2*A*⋯O4^iii^	0.89	1.95	2.8146 (14)	165
N2—H2*B*⋯O*W*1^v^	0.89	1.87	2.7574 (17)	172
N2—H2*C*⋯O4^ii^	0.89	1.91	2.7918 (14)	171

Symmetry codes: (i) 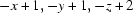; (ii) 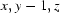; (iii) 
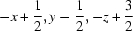
; (iv) 
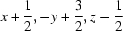
; (v) 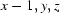.

**Fig. 1.**
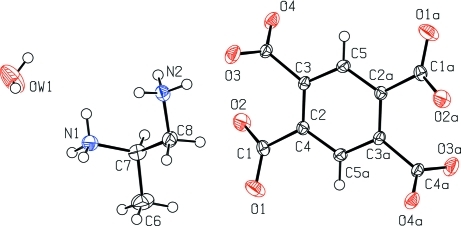
The molecular structure of the title molecule, with the atom-numbering scheme. Displacement ellipsoids are drawn at the 30% probability level.

**Fig. 2.**
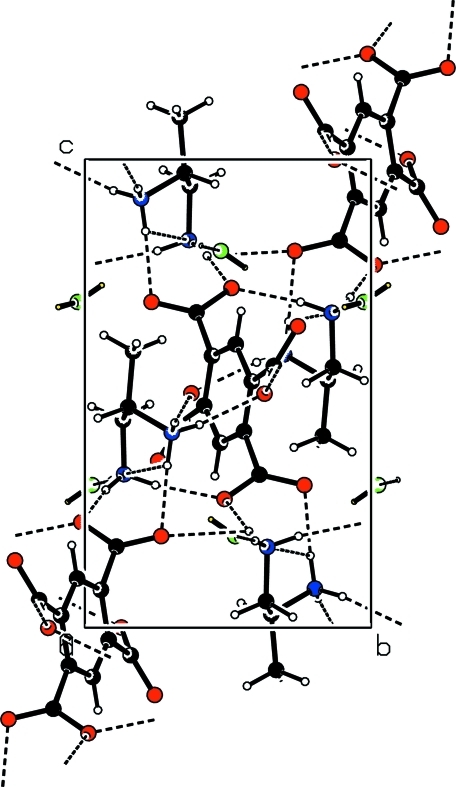
Crystal packing if the title compound viewed down the a axis. Dashed lines indicate N—H···O and O—H···O hydrogen bonding interactions involving cation, anion and water species forming a supermolecular 3-D structure.

## supplementary crystallographic information

### Comment

Several proton transfer systems using benzene- 1,2,4,5-tetracarboxylic acid (H_4_BTC), with nitrogen donor molecules, such as 1,10-phenanthroline, 1,7-phenanthroline, phenazine, 4-(*N*,*N*-dimethylamino)pyridine, 1,2-bis(4-pyridyl) ethene and 1,2-bis(4-pyridyl)ethane, have been synthesized and characterized by X-ray diffraction methods (Aghabozorg *et al.* 2008, Arora & Pedireddi, 2003). R%elated crystal structures of (H_2_BTC)(Hphen)2- (H_4_BTC) (where phen is 1,10-phenanthroline; Wang *et al.*, 2005), (H_3_BTC)(Hbipy).3H2O (where bipy is 2,20-bipyridine; Ma *et al.*, 2005), (H_2_BTC)(Hbipy)_2_(H_4_BTC) (Mrvos-Sermek *et al.*, 1996) and (BTC)(H_2_en)_2_.2H_2_O (Rafizadeh *et al.* 2006) have been reported previously.

In the title compound, (C_3_H_12_N_2_)_2_^2+^ (C_10_H_2_O_8_)^4-^. 2H_2_O, the asymmetric unit contains two diprotonated propane-1,2-diamine cations, one tetraanionic deprotonated anion form of benzene-1,2,4,5-tetracarboxylic acid and two water molecules (Fig. 1).  The dication forms from the transfer of a single proton from each of the carboxyl groups to a propane-1,2-diamine molecule.  The negative charges of one of the tetraanionic 1,2,4,5-benzenetetracarboxylate groups, is thereby neutralized by a doubly protonated propane-1,2-diammonium fragment. Crystal packing is stabilized by extensive N—H···O and O—H···O hydrogen bonding interactions involving all three species forming a supramolecular 3-D structure (Fig. 2).

### Experimental

Propane-1,2-diamine (12.6 mmol) was added to a solution of benzene-1,2,4,5-tetracarboxylic acid (1.65 g, 6.3 mmol) in ethanol (30 ml) at room temperature. The milky precipitated product was recrystallized from water. After one week, colorless plate crystals of (I) were isolated (yield 87.1%, decomposition < 420 K)

### Refinement

All of the H atoms were placed in their calculated positions and then refined using the riding model with Atom—H lengths of 0.93, 0.98Å (CH), 0.97Å (CH_2_) 0.96Å (CH_3_) or 0.89Å (NH). Isotropic displacement parameters for these atoms were set to 1.5 times (NH), 1.2 (CH, CH_2_, CH_3_) times *U*_eq_ of the parent atom.  Water H atoms atoms were located in a difference Fourier map and refined isotropically without restraint.

### Crystal data

**Table:**

2C_3_H_12_N_2_^2+^·C_10_H_2_O_8_^4^^−^·2H_2_O	*F*(000) = 468
*M**_r_* = 438.44	*D*_x_ = 1.441 Mg m^−^^3^
Monoclinic, *P*2_1_/*n*	Mo *K*α radiation, λ = 0.71073 Å
Hall symbol: -P 2yn	Cell parameters from 8157 reflections
*a* = 10.427 (2) Å	θ = 2.3–30.5°
*b* = 7.6955 (15) Å	µ = 0.12 mm^−^^1^
*c* = 12.854 (3) Å	*T* = 298 K
β = 101.61 (3)°	Plate, colorless
*V* = 1010.3 (3) Å^3^	0.49 × 0.40 × 0.08 mm
*Z* = 2	

### Data collection

**Table:**

Bruker SMART CD area-detector diffractometer	3038 independent reflections
Radiation source: fine-focus sealed tube	2727 reflections with *I* > 2σ(*I*)
graphite	*R*_int_ = 0.026
φ and ω scans	θ_max_ = 30.5°, θ_min_ = 2.3°
Absorption correction: multi-scan (*SADABS*; Sheldrick, 1998)	*h* = −14→14
*T*_min_ = 0.940, *T*_max_ = 0.990	*k* = −10→10
8157 measured reflections	*l* = −15→18

### Refinement

**Table:**

Refinement on *F*^2^	Primary atom site location: structure-invariant direct methods
Least-squares matrix: full	Secondary atom site location: difference Fourier map
*R*[*F*^2^ > 2σ(*F*^2^)] = 0.041	Hydrogen site location: inferred from neighbouring sites
*wR*(*F*^2^) = 0.112	H atoms treated by a mixture of independent and constrained refinement
*S* = 1.07	*w* = 1/[σ^2^(*F*_o_^2^) + (0.0571*P*)^2^ + 0.3092*P*] where *P* = (*F*_o_^2^ + 2*F*_c_^2^)/3
3038 reflections	(Δ/σ)_max_ = 0.004
146 parameters	Δρ_max_ = 0.39 e Å^−^^3^
0 restraints	Δρ_min_ = −0.19 e Å^−^^3^

### Special details

**Table:**

Geometry. All e.s.d.'s (except the e.s.d. in the dihedral angle between two l.s. planes) are estimated using the full covariance matrix. The cell e.s.d.'s are taken into account individually in the estimation of e.s.d.'s in distances, angles and torsion angles; correlations between e.s.d.'s in cell parameters are only used when they are defined by crystal symmetry. An approximate (isotropic) treatment of cell e.s.d.'s is used for estimating e.s.d.'s involving l.s. planes.
Refinement. Refinement of *F*^2^ against ALL reflections. The weighted *R*-factor *wR* and goodness of fit *S* are based on *F*^2^, conventional *R*-factors *R* are based on *F*, with *F* set to zero for negative *F*^2^. The threshold expression of *F*^2^ > σ(*F*^2^) is used only for calculating *R*-factors(gt) *etc*. and is not relevant to the choice of reflections for refinement. *R*-factors based on *F*^2^ are statistically about twice as large as those based on *F*, and *R*- factors based on ALL data will be even larger.

### Fractional atomic coordinates and isotropic or equivalent isotropic displacement parameters (Å^2^)

**Table:**

	*x*	*y*	*z*	*U*_iso_*/*U*_eq_	
C1	0.25868 (9)	0.83996 (13)	1.05876 (8)	0.01914 (19)	
C2	0.12279 (9)	0.91697 (12)	1.02583 (7)	0.01626 (18)	
C3	0.06315 (9)	0.94408 (12)	0.91926 (7)	0.01595 (17)	
C4	0.12422 (9)	0.88991 (13)	0.82686 (7)	0.01717 (18)	
C5	−0.05822 (9)	1.02713 (13)	0.89486 (7)	0.01784 (18)	
H5	−0.0971	1.0457	0.8240	0.021*	
C6	0.39782 (14)	0.3316 (2)	1.09027 (10)	0.0389 (3)	
H6A	0.3587	0.2308	1.1150	0.047*	
H6B	0.3728	0.4332	1.1246	0.047*	
H6C	0.4914	0.3200	1.1067	0.047*	
C7	0.35129 (11)	0.34862 (15)	0.97084 (9)	0.0245 (2)	
H7	0.3899	0.4541	0.9475	0.029*	
C8	0.20338 (10)	0.36693 (14)	0.94363 (9)	0.0241 (2)	
H8A	0.1645	0.2735	0.9774	0.029*	
H8B	0.1789	0.4758	0.9723	0.029*	
N1	0.39794 (8)	0.19628 (13)	0.91649 (7)	0.02395 (19)	
H1A	0.3594	0.1001	0.9333	0.036*	
H1B	0.4843	0.1861	0.9373	0.036*	
H1C	0.3779	0.2120	0.8465	0.036*	
N2	0.14955 (9)	0.36274 (12)	0.82766 (7)	0.02209 (18)	
H2A	0.1997	0.4268	0.7942	0.033*	
H2B	0.0685	0.4053	0.8147	0.033*	
H2C	0.1481	0.2536	0.8046	0.033*	
O1	0.28549 (8)	0.75595 (13)	1.14363 (7)	0.0344 (2)	
O2	0.33872 (7)	0.87232 (12)	0.99958 (7)	0.0302 (2)	
O3	0.12767 (9)	0.73323 (10)	0.80446 (7)	0.02951 (19)	
O4	0.16276 (8)	1.01227 (10)	0.77470 (6)	0.02490 (18)	
OW1	0.89422 (11)	0.47495 (18)	0.80452 (13)	0.0566 (4)	
H1W	0.833 (2)	0.408 (3)	0.8154 (16)	0.052 (5)*	
H2W	0.869 (2)	0.559 (3)	0.769 (2)	0.065 (7)*	

### Atomic displacement parameters (Å^2^)

**Table:**

	*U*^11^	*U*^22^	*U*^33^	*U*^12^	*U*^13^	*U*^23^
C1	0.0145 (4)	0.0196 (4)	0.0234 (4)	0.0020 (3)	0.0040 (3)	0.0006 (3)
C2	0.0141 (4)	0.0176 (4)	0.0179 (4)	0.0015 (3)	0.0053 (3)	0.0012 (3)
C3	0.0154 (4)	0.0171 (4)	0.0167 (4)	0.0003 (3)	0.0067 (3)	−0.0010 (3)
C4	0.0160 (4)	0.0199 (4)	0.0167 (4)	0.0015 (3)	0.0059 (3)	−0.0016 (3)
C5	0.0161 (4)	0.0230 (4)	0.0148 (4)	0.0020 (3)	0.0040 (3)	0.0008 (3)
C6	0.0414 (7)	0.0510 (8)	0.0217 (5)	−0.0021 (6)	0.0002 (5)	−0.0028 (5)
C7	0.0240 (5)	0.0262 (5)	0.0229 (5)	−0.0040 (4)	0.0038 (4)	−0.0001 (4)
C8	0.0241 (5)	0.0253 (5)	0.0243 (5)	0.0013 (4)	0.0086 (4)	−0.0007 (4)
N1	0.0187 (4)	0.0302 (5)	0.0234 (4)	0.0020 (3)	0.0052 (3)	0.0036 (3)
N2	0.0202 (4)	0.0208 (4)	0.0257 (4)	0.0013 (3)	0.0058 (3)	0.0023 (3)
O1	0.0243 (4)	0.0431 (5)	0.0355 (5)	0.0087 (3)	0.0055 (3)	0.0183 (4)
O2	0.0167 (3)	0.0416 (5)	0.0345 (4)	0.0056 (3)	0.0107 (3)	0.0097 (4)
O3	0.0440 (5)	0.0192 (4)	0.0302 (4)	−0.0002 (3)	0.0189 (3)	−0.0043 (3)
O4	0.0322 (4)	0.0211 (4)	0.0262 (4)	−0.0002 (3)	0.0174 (3)	0.0001 (3)
OW1	0.0266 (5)	0.0496 (7)	0.0948 (10)	0.0052 (5)	0.0151 (5)	0.0303 (7)

### Geometric parameters (Å, °)

**Table:**

O1—C1	1.2504 (14)	C1—C2	1.5155 (14)
O2—C1	1.2620 (13)	C2—C5^i^	1.3978 (13)
O3—C4	1.2418 (13)	C2—C3	1.4014 (13)
O4—C4	1.2673 (13)	C3—C5	1.3959 (14)
OW1—H1W	0.85 (2)	C3—C4	1.5147 (13)
OW1—H2W	0.81 (2)	C5—H5	0.9300
N1—C7	1.4953 (15)	C6—C7	1.5197 (17)
N2—C8	1.4837 (15)	C7—C8	1.5178 (16)
N1—H1B	0.8900	C6—H6B	0.9600
N1—H1C	0.8900	C6—H6C	0.9600
N1—H1A	0.8900	C6—H6A	0.9600
N2—H2C	0.8900	C7—H7	0.9800
N2—H2B	0.8900	C8—H8B	0.9700
N2—H2A	0.8900	C8—H8A	0.9700
			
O1···OW1^ii^	2.7596 (17)	C3···H8A^vi^	2.7900
O1···OW1^iii^	2.9845 (19)	C4···H2C^vi^	2.8300
OW1···O1^iv^	2.9845 (19)	C4···H1A^vi^	3.0300
OW1···O3^v^	3.1427 (17)	C4···H2A^ix^	2.6500
OW1···N2^v^	2.7574 (17)	C4···H1C^ix^	2.6100
OW1···O1^ii^	2.7596 (17)	C5···H8A^vi^	3.0200
O2···C4	2.8198 (14)	C6···H7^ii^	2.8800
O2···O3	3.1713 (14)	C7···H7^ii^	3.0900
O2···N1^vi^	2.8289 (15)	H1W···H2B^v^	2.4600
O2···N1^ii^	2.7910 (13)	H1W···O1^ii^	1.91 (2)
O3···C8	3.3468 (16)	H1W···C1^ii^	2.79 (2)
O3···O2	3.1713 (14)	H1A···C4^xii^	3.0300
O3···C1	3.3806 (16)	H1A···O2^xii^	1.9800
O3···N2	2.8710 (14)	H1A···H6A	2.5400
O3···OW1^vii^	3.1427 (17)	H1A···H8A	2.5900
O3···O4^viii^	3.0979 (14)	H1A···C1^xii^	2.9000
O3···N1^ix^	2.8130 (14)	H1A···O4^xii^	2.6700
O4···N2^vi^	2.7918 (14)	H1B···O1^ii^	2.8400
O4···N2^ix^	2.8146 (14)	H1B···C1^ii^	2.6800
O4···N1^vi^	3.0889 (14)	H1B···O2^ii^	1.9100
O4···C7^ix^	3.3738 (16)	H1B···H6C	2.4000
O4···O3^ix^	3.0979 (14)	H1C···O4^xii^	2.7200
O1···H1B^ii^	2.8400	H1C···C4^viii^	2.6100
O1···H6B	2.6700	H1C···O3^viii^	1.9400
O1···H5^i^	2.5900	H1C···O4^viii^	2.7700
O1···H2W^iii^	2.20 (2)	H1C···H2A	2.4800
O1···H1W^ii^	1.91 (2)	H1C···H2C	2.3700
OW1···H6A^x^	2.8700	H1C···N2	2.6200
OW1···H2B^v^	1.8700	H2W···C1^iv^	2.83 (2)
O2···H1A^vi^	1.9800	H2W···H2B^v^	2.3600
O2···H6C^ii^	2.8600	H2W···O1^iv^	2.20 (2)
O2···H1B^ii^	1.9100	H2A···O4^viii^	1.9500
O3···H8B	2.9000	H2A···N1	2.9300
O3···H2A^ix^	2.8300	H2A···O3	2.4900
O3···H1C^ix^	1.9400	H2A···C4^viii^	2.6500
O3···H2A	2.4900	H2A···O3^viii^	2.8300
O3···H2B	2.6100	H2A···H1C	2.4800
O4···H2A^ix^	1.9500	H2A···H7	2.5100
O4···H1C^ix^	2.7700	H2B···O3	2.6100
O4···H5	2.9200	H2B···H2W^vii^	2.3600
O4···H7^ix^	2.8300	H2B···OW1^vii^	1.8700
O4···H6C^xi^	2.8200	H2B···H1W^vii^	2.4600
O4···H2C^vi^	1.9100	H2C···C3^xii^	3.0300
O4···H1A^vi^	2.6700	H2C···N1	2.7500
O4···H1C^vi^	2.7200	H2C···H1C	2.3700
N1···N2	2.9090 (15)	H2C···O4^xii^	1.9100
N1···O2^xii^	2.8289 (15)	H2C···C4^xii^	2.8300
N1···O4^xii^	3.0889 (14)	H5···O1^i^	2.5900
N1···C4^viii^	3.4304 (15)	H5···H6B^xi^	2.5200
N1···O2^ii^	2.7910 (13)	H5···O4	2.9200
N1···O3^viii^	2.8130 (14)	H6A···OW1^xiii^	2.8700
N2···O4^viii^	2.8146 (14)	H6A···H1A	2.5400
N2···O3	2.8710 (14)	H6A···H8A	2.4300
N2···O4^xii^	2.7918 (14)	H6B···H5^xiv^	2.5200
N2···N1	2.9090 (15)	H6B···H8B	2.5400
N2···C4^viii^	3.3822 (15)	H6B···O1	2.6700
N2···OW1^vii^	2.7574 (17)	H6C···O2^ii^	2.8600
N1···H2C	2.7500	H6C···H7^ii^	2.3200
N1···H2A	2.9300	H6C···H1B	2.4000
N2···H1C	2.6200	H6C···O4^xiv^	2.8200
C1···O3	3.3806 (16)	H7···H6C^ii^	2.3200
C3···C8^vi^	3.5554 (16)	H7···H7^ii^	2.5200
C4···N1^ix^	3.4304 (15)	H7···C7^ii^	3.0900
C4···O2	2.8198 (14)	H7···H2A	2.5100
C4···N2^ix^	3.3822 (15)	H7···O4^viii^	2.8300
C7···O4^viii^	3.3738 (16)	H7···C6^ii^	2.8800
C8···O3	3.3468 (16)	H8A···H6A	2.4300
C8···C3^xii^	3.5554 (16)	H8A···C3^xii^	2.7900
C1···H1B^ii^	2.6800	H8A···C5^xii^	3.0200
C1···H2W^iii^	2.83 (2)	H8A···C2^xii^	2.8700
C1···H1A^vi^	2.9000	H8A···H1A	2.5900
C1···H8B	3.0700	H8B···C1	3.0700
C1···H1W^ii^	2.79 (2)	H8B···H6B	2.5400
C2···H8A^vi^	2.8700	H8B···O3	2.9000
C3···H2C^vi^	3.0300		
			
H1W—OW1—H2W	114 (2)	O3—C4—O4	124.87 (10)
H1A—N1—H1C	109.00	O4—C4—C3	116.01 (9)
H1B—N1—H1C	109.00	C2^i^—C5—C3	121.67 (9)
C7—N1—H1C	109.00	C3—C5—H5	119.00
C7—N1—H1A	109.00	C2^i^—C5—H5	119.00
C7—N1—H1B	109.00	C6—C7—C8	110.11 (10)
H1A—N1—H1B	109.00	N1—C7—C6	109.67 (10)
C8—N2—H2A	109.00	N1—C7—C8	112.09 (9)
H2A—N2—H2C	110.00	N2—C8—C7	113.01 (9)
C8—N2—H2C	109.00	C7—C6—H6B	109.00
H2A—N2—H2B	109.00	C7—C6—H6C	110.00
C8—N2—H2B	109.00	H6A—C6—H6B	109.00
H2B—N2—H2C	109.00	H6A—C6—H6C	109.00
O2—C1—C2	116.68 (9)	H6B—C6—H6C	109.00
O1—C1—O2	124.68 (10)	C7—C6—H6A	109.00
O1—C1—C2	118.57 (9)	N1—C7—H7	108.00
C1—C2—C3	122.50 (9)	C8—C7—H7	108.00
C3—C2—C5^i^	118.88 (9)	C6—C7—H7	108.00
C1—C2—C5^i^	118.50 (9)	N2—C8—H8A	109.00
C2—C3—C4	123.48 (9)	N2—C8—H8B	109.00
C2—C3—C5	119.45 (9)	C7—C8—H8B	109.00
C4—C3—C5	117.07 (8)	H8A—C8—H8B	108.00
O3—C4—C3	119.04 (9)	C7—C8—H8A	109.00
			
O1—C1—C2—C5^i^	32.71 (14)	C5^i^—C2—C3—C5	0.53 (14)
O2—C1—C2—C3	31.49 (14)	C5—C3—C4—O3	−107.87 (11)
O1—C1—C2—C3	−151.38 (10)	C4—C3—C5—C2^i^	−179.74 (9)
O2—C1—C2—C5^i^	−144.42 (10)	C5—C3—C4—O4	68.84 (12)
C1—C2—C3—C4	3.77 (15)	C2—C3—C5—C2^i^	−0.54 (15)
C1—C2—C3—C5	−175.37 (9)	C2—C3—C4—O4	−110.32 (11)
C3—C2—C5^i^—C3^i^	−0.54 (15)	C2—C3—C4—O3	72.98 (13)
C1—C2—C5^i^—C3^i^	175.52 (9)	N1—C7—C8—N2	−50.66 (12)
C5^i^—C2—C3—C4	179.67 (9)	C6—C7—C8—N2	−173.04 (10)

Symmetry codes: (i) −*x*, −*y*+2, −*z*+2; (ii) −*x*+1, −*y*+1, −*z*+2; (iii) *x*−1/2, −*y*+3/2, *z*+1/2; (iv) *x*+1/2, −*y*+3/2, *z*−1/2; (v) *x*+1, *y*, *z*; (vi) *x*, *y*+1, *z*; (vii) *x*−1, *y*, *z*; (viii) −*x*+1/2, *y*−1/2, −*z*+3/2; (ix) −*x*+1/2, *y*+1/2, −*z*+3/2; (x) *x*+1/2, −*y*+1/2, *z*−1/2; (xi) *x*−1/2, −*y*+3/2, *z*−1/2; (xii) *x*, *y*−1, *z*; (xiii) *x*−1/2, −*y*+1/2, *z*+1/2; (xiv) *x*+1/2, −*y*+3/2, *z*+1/2.

### Hydrogen-bond geometry (Å, °)

**Table:**

*D*—H···*A*	*D*—H	H···*A*	*D*···*A*	*D*—H···*A*
OW1—H1W···O1^ii^	0.85 (2)	1.91 (2)	2.7596 (17)	172 (2)
N1—H1A···O2^xii^	0.89	1.98	2.8289 (15)	159
N1—H1B···O2^ii^	0.89	1.91	2.7910 (13)	168
N1—H1C···O3^viii^	0.89	1.94	2.8130 (14)	168
OW1—H2W···O1^iv^	0.81 (2)	2.20 (2)	2.9845 (19)	167 (2)
N2—H2A···O4^viii^	0.89	1.95	2.8146 (14)	165
N2—H2B···OW1^vii^	0.89	1.87	2.7574 (17)	172
N2—H2C···O4^xii^	0.89	1.91	2.7918 (14)	171

Symmetry codes: (ii) −*x*+1, −*y*+1, −*z*+2; (xii) *x*, *y*−1, *z*; (viii) −*x*+1/2, *y*−1/2, −*z*+3/2; (iv) *x*+1/2, −*y*+3/2, *z*−1/2; (vii) *x*−1, *y*, *z*.
